# Revisiting the Physicochemical Properties and Applications of Deep Eutectic Solvents

**DOI:** 10.3390/molecules27041368

**Published:** 2022-02-17

**Authors:** Sushma P. Ijardar, Vickramjeet Singh, Ramesh L. Gardas

**Affiliations:** 1 Department of Chemistry, Veer Narmad South Gujarat University, Surat 395007, India; sijardar@vnsgu.ac.in; 2 Department of Chemistry, Dr. B. R. Ambedkar National Institute of Technology, Jalandhar 144011, India; singhvj@nitj.ac.in; 3 Department of Chemistry, Indian Institute of Technology Madras, Chennai 600036, India

**Keywords:** deep eutectic solvents, physicochemical properties, applications

## Abstract

Recently, deep eutectic solvent (DES) or ionic liquid (IL) analogues have been considered as the newest green solvent, demonstrating the potential to replace harsh volatile organic solvents. DESs are mainly a combination of two compounds: hydrogen bond acceptor (HBA) and hydrogen bond donor (HBD), which have the ability to interact through extensive hydrogen bonds. A thorough understanding of their physicochemical properties is essential, given their successful applications on an industrial scale. The appropriate blend of HBA to HBD can easily fine-tune DES properties for desired applications. In this context, we have reviewed the basic information related to DESs, the two most studied physicochemical properties (density and viscosity), and their performance as a solvent in (i) drug delivery and (ii) extraction of biomolecules. A broader approach of various factors affecting their performance has been considered, giving a detailed picture of the current status of DESs in research and development.

## 1. Introduction

Solvents have been utilized for a number of applications, ranging from drug delivery/discovery to extraction of biologically important compounds. Typically volatile organic solvents, have shown tremendous potential for these targeted applications, but they suffer from adverse effects related to low volatility and toxicity, and they may cause harm to the environment. In this regard, the growing interest of chemical industries and the research communities in “green and sustainable solvents” is attributed to incorporating 12 green principles. A significant portion of harsh solvents have been used in multiple applications, i.e., reaction, solubilization, precipitation, and separation. Such solvent systems can contribute considerably to environmental pollution [1,2]. Several benign solvents such as water, polyethylene glycol, ethyl lactate, perfluorinated solvents, and supercritical fluids have been proposed, as well as new solvents such as deep eutectic solvents (DESs) and ionic liquids (ILs), have been developed in the past two decades to minimize the quality-of-air effects of volatile organic solvents [1,2,3,4].

As in the literature, ionic liquids were studied as the most promising green solvents during the last two decades [3,4]. ILs are a group of molten salts that were initially prepared by combining imidazolium cation with different anions tetrafluoroborate (BF_4_), hexafluorophosphate (PF_6_), chloride (Cl), and bis(trifluoromethane)sulfonimide (NTf_2_). The structural variance in cation moiety and a wide choice of anions for the preparation of ILs give a broad spectrum of ILs with different physicochemical properties [5,6]. Theoretically, there are 10^18^ kinds of different combinations for ILs [7]. They have been utilized successfully as solvents in widespread applications such as in separation processes, chemical manufacturing processes, polymer synthesis, catalysis, membrane technology extraction, biomass processing, and batteries and energy applications, to name a few [8,9,10,11,12]. The considerable utilization of ILs is credited to their variety of physicochemical properties such as no or little room temperature vapor pressure, chemical and thermal stability, higher solubility and conductivity, broad electrochemical windows and liquid range, and the possibility of recycling [6,8,13,14].

Despite their huge success, ILs have been scrutinized for greener credentials, toxicity, higher cost of starting materials, complex synthesis procedure, difficulty in purification, and regeneration cycling [15]. The purity of ILs is of highest importance, as traces of impurity can alter the physicochemical properties of pure ILs. The synthesis of ILs also produces a large number of solvents and salts during ion exchange [16]. New solvents are needed to justify the green solvent tag, and which can replace or overcome the limitations associated with ILs. In this regard, “Deep Eutectic Solvents” have been introduced recently. DESs are less toxic, highly degradable, biocompatible, and easily prepared from the readily available starting material. DESs share the same physicochemical properties; hence, they are known as “ILs analogues”. Though DESs are non-ionic species, they can be obtained by combining suitable hydrogen bond donors and hydrogen bond acceptors, for instance, quaternary ammonium salts and metal salts [17].

DESs can be obtained by mixing two components to form a eutectic mixture. Choline chloride (ChCl), quaternary ammonium salts are amongst the most studied components used for the preparation of DESs. ChCl can readily form a eutectic mixture with urea, carbohydrates, amino acids, and carboxylic acids via hydrogen bonding. DESs have unique characteristics: (i) they are designed in numerous combinations of two readily biodegradable compounds, HBA (hydrogen bond acceptor) and HBD (hydrogen bond donor), in an appropriate molar ratio; (ii) they are cheaper; (iii) they do not need purification; and (iv) they are biocompatible and biodegradable. For these reasons, considerable growth has been observed in the designing and applications of DESs [18,19,20,21].

DESs have been considered superior solvents to several traditional ILs for the following reasons. (i) Ease of preparation: the preparation of most ILs is complex compared to the preparation of DESs, which is prepared quickly by a simple blending of two components at moderate temperature. This involves lower production value with respect to conventional ILs and permits large scale applications. The preparation of DESs can be obtained with nearly 100% atom economy. (ii) Easy availability of components of DESs: ILs are generally expensive and often toxic and non-biodegradable. In the case of DESs, quaternary ammonium/phosphonium/sulfonium salts and various organic compounds used as HBDs are readily available, as they are produced in vast amounts at a lower cost than the conventional reagents. The components of DESs are less toxic to be scaled up for large-scale production [22,23,24].

## 2. Deep Eutectic Solvents

### 2.1. Definition

**Figure 1:**
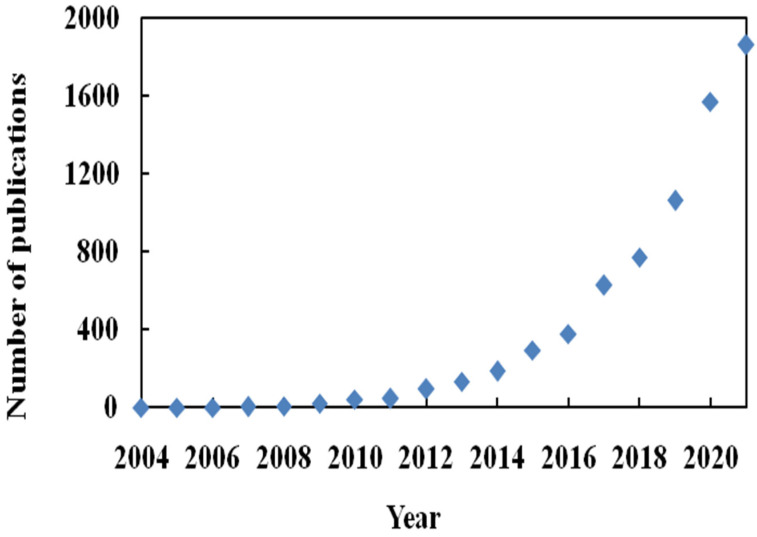
Number of publications per year for term deep eutectic solvents.

Generally, DESs are a highly non-ideal mixture of two biodegradable components (HBA and HBD) associated with strong hydrogen bonding interactions. The resulting liquid will have a melting point lower than the melting points of both HBA and HBD. They are broadly defined as liquid produced due to greater depression in the freezing point. Most DESs are a combination of quaternary ammonium salt, as HBA and HBD have the ability to form hydrogen bonding with HBA [2]. DESs were first introduced in 2003 by Abbott et al. for the mixture of ChCl (HBA) and urea (HBD) [17]. They showed that clear, deep eutectic liquid formed when ChCl with a melting point of 302 °C and urea with a melting point of 133 °C are mixed together in a definite molar ratio. The melting points of DESs were found to decrease to very low levels, around 12 °C, which is lower than the melting point of the two mixing compounds. According to this definition, many DESs have been prepared and applied as a solvent in organic reactions, extraction of dyes, protein, nucleic acids, metals, azeotropic separation, and more [18,19,20,21]. The list of HBAs and HBDs commonly used to prepare DESs is listed in Figure 1. The SciFinder analysis dated 31st December 2021 displayed 7226 publications for the term “Deep Eutectic Solvents”. It is evident from Figure 1 that the maximum publications were reported in 2021, and research in the subject of DESs is continuously growing.

### 2.2. Method of Preparation

As discussed in the introduction, DESs are prepared by 100% atom economy route, as it involves simple mixing of HBA and HBD, and all other steps such as purification and waste disposal were eliminated or not required. (i) The most common preparation method, HBA and HBD of DESs, were heated and continuously stirred together in an inert atmosphere until homogeneous liquids were formed [17]. (ii) In the second approach, the evaporating method, the DES components were first dissolved in water. The water was then evaporated under vacuum at 323 K. The resultant mixture was kept in the desiccator until the attainment of stable weight. (iii) In the grinding method, solid HBA and HBD were added to a mortar kept in a glove box under an inert nitrogen atmosphere and ground continuously until a homogeneous transparent liquid was formed [25]. (iv) In the freeze-drying technique, HBA and HBD were first dissolved in water (5 wt%). The two aqueous solutions are mixed and then frozen. After that, the mixture was kept freeze-dried to obtain homogeneous and clear liquid [26].

### 2.3. Types of DES

DESs were presented using the general formula: R^+^A^−^*x*B, where R^+^ is ammonium, sulfonium, and phosphonium cation core. A and B are Lewis base with halide anion and Levis acid, respectively [20]. The complex formation is observed between x and Lewis or Brønsted acid B (x defined as B number of molecules reacted with anion). DESs are mainly classified based upon the nature of the HBD used, as shown in Table 1. Four main types of DESs have been reported. The possibility of a fifth type of DES has also been reported, but not enough literature about it is available yet.

**Table 1:** Classification of DESs.

Type of DES	General Formula	Terms	Example
I	R^+^ A^−^ + cMCl_x_	M = In, Zn, Fe, Al, Sn	ChCl + SnCl_2_
II	R^+^A^−^ + cMCl_x_.cH_2_O	M = Ni, Cr, Fe, Cu	ChCl + FeCl_3_·6H_2_O
III	R^+^ A^−^ + cRW	W = OH, CONH_2,_ COOH	ChCl + Urea
IV	MCl_x_ + cRW	M = Al, Zn and W = CONH_2_, OH	ZnCl_2_ + Urea
V	HBD + HBA	HBD = hydrogen bond donor HBA = hydrogen bond acceptor	Thymol + Menthol

Type I DESs: This type of DES can be prepared from quaternary ammonium salt and metal chloride. Type I DESs formed with imidazolium salts and various metal halides such as ZnCl_2_, FeCl_2_, AgCl, CuCl_2_, CdCl_2_, LiCl, SnCl_2,_ and SnCl_4_ [27,28]. The low melting point non-hydrated metal halides used to form type I DESs are very few; hence, fewer HBD combinations are available for this type of DES.

Type II DESs: This type of DES can be composed of quaternary ammonium salt and hydrate of metal chloride hydrate [29].

**Figure 2:**
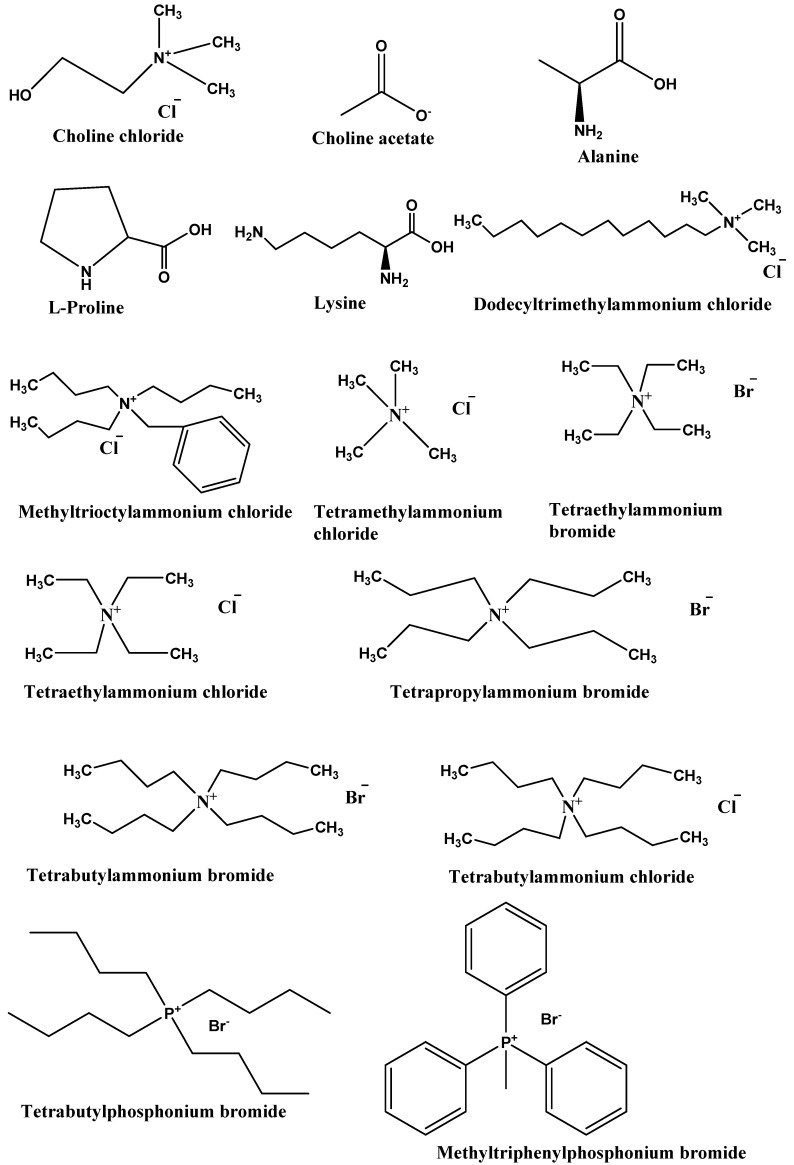
List of commonly used HBAs in preparation of DESs.

**Figure 3:**
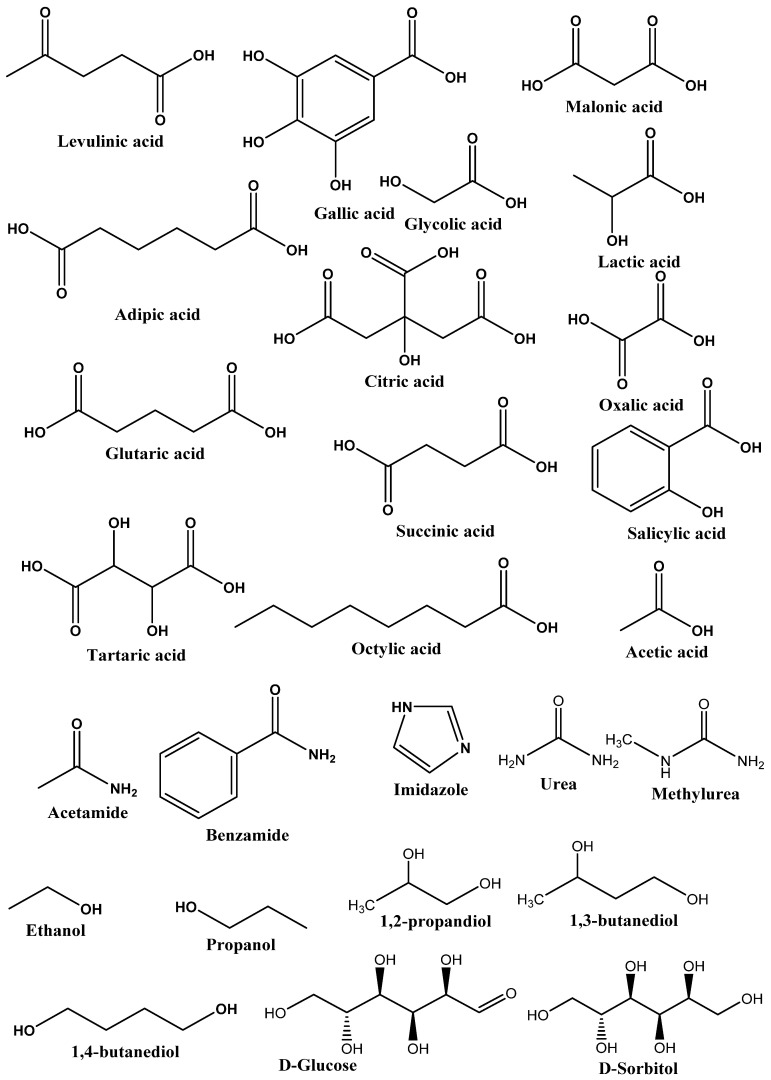
List of commonly used HBDs in preparation of DESs.

Type III DESs: This type of DES can be prepared using quaternary ammonium salt as HBA and HBD. This type of DES is widely studied. These DESs are mainly composed of choline chloride and HBDs (carboxylic acids, alcohols, amides, and carbohydrates, etc.) The HBA used in the preparation of this kind of DES are listed in Figure 2. These types of DESs are the most important due to their ability to solvate a wide range of transition metal species [17]. These solvents are simple to prepare, less expensive, relatively unreactive with water, and many are biodegradable. A wide range of HBDs (Figure 3) are available for the preparation of this class of DES. The physical properties of this type of DES are dependent on the nature of the hydrogen bond donor and can be customized easily for any given application.

Type IV DESs: These types of DESs are composed of metal chloride (particularly transition metal chloride) and HBD. It was reported to form a DES using a mixture of ZnCl_2_ and urea. In non-aqueous solvents, these metal salts usually do not ionize but form a DES with ethylene glycol, acetamide, and 1,6-hexanediol [29,30].

Type V DESs: These types of DESs are a relatively new class mixture of non-ionic molecular HBA and HBD [31]. Although it exhibits depression in melting points like DESs, there is no ionic contribution. Hydrogen bonding was especially predominant in this class of DESs. There is a possibility of another new class of mixture that does not fit precisely into this category. However, they exhibit deep depression in melting points like a mixture of Brønsted or Lowry acids:bases.

## 3. Physicochemical Properties of DESs

Like ILs, DESs are also characterized by a collection of unique physicochemical properties: DESs have been used as potential solvents in the industry, owing to their numerous physicochemical properties such as density, freezing temperature, viscosity, surface tension, miscibility, conductivity, and polarity, etc. Moreover, a huge number of DESs can be obtained using different combinations of HBA and HBD, making DESs more designable, thus named designer solvents. The unique combination of HBA:HBD resulted in DESs with different physicochemical properties. The physicochemical properties of DESs can be manipulated using various combinations of HBA and HBD in different molar ratios. Apart from the variety of HBA to HBD, the physicochemical properties of DESs are also affected by the molar ratio of HBA and HBD, purity of HBA and HBD, temperature, water content, and method of preparation [24].

The determination of physicochemical properties of DESs is in the framework of their applications as a solvent in chemical processes. The design of DESs for specific applications needs knowledge of the physicochemical properties of DESs. Importantly, the accurate knowledge of physicochemical properties such as density, speed of sound, refractive index, and viscosity of DESs are crucial for their industrial exposure as a solvent in various operation unit processes [32,33]. Hence, the number of publications for physicochemical properties are continuously increasing. The first publication was reported by Abbott et al. in 2011, and in 2021, around 221 publications exclusively reported physicochemical properties of various DESs.

### 3.1. Density

The PVT data of densities of DESs are crucial parameters for designing equipment and processes, liquid–liquid equilibria, mass transfer, the development of equations of state and predicting models, and the calculation of thermodynamic properties such as viscosity, expansion coefficient, and isothermal compressibility [34]. The densities of various DESs are shown in Table 2 to indicate the change in density of DESs with respect to change in HBD, molar ratio of HBA:HBD, and method of preparation. The literature reveals that most DES densities are higher than the density of water, between 1.0 and 1.35 gcm^3^ at 298.15 K [21]. The values mostly vary with the change in combinations of HBA to HBD. DESs composed of common HBA ZnCl_2_ and different HBDs have densities between 1.3 to 1.6 g cm^−3^ [29]. Similarly, the density of DESs composed of ChCl and various acids as HBDs were reported between 1.0 to 1.6 g cm^−3^ [35].

The difference in DES density with respect to various factors can be understood using the Hole theory [36]. The Hole theory provides linkage between properties of DESs and available holes of approximate dimension and size of mobile species. DESs are assumed as a composition of holes. During the formation of DESs, when HBD were added to HBA, the average hole size of holes was changed, hence changing the density of the DES. Similarly, one can say the change in DES density can result from dynamics of molecular interactions and available free volumes in the DES. In the case of ZnCl_2_:urea, the average radius of Hole was found to reduce, resulting in a small increase in density value of a DES composed of ChCl:urea [29]. There are other factors that also govern the actual density of DESs, which can be seen in Table 2 [37,38,39,40,41,42,43,44,45,46,47,48,49,50,51,52,53,54,55,56,57,58,59,60,61,62,63,64,65] and are also listed below:

(1) Nature of HBD: The densities of DESs obviously depend upon the nature of the HBD [25,29,33,37,38,42,43,45,47,48,49,50,51,52,55,64]. For example, Florindo et al. have studied DESs composed of ChCl:carboxylic acids (oxalic acid, glycolic acid, malonic acid, glutaric acid, and levulinic acid) of different alkyl chain length. The densities of DESs composed of glutaric acid and levulinic acid were less than those of DESs of other acids. The presence of a long alkyl chain of C_5_ was the main reason for the lower density. Again, DESs composed of levulinic acid have a lower density than those of glutaric acid due to higher moles of acid (1:2) in the DES. The densities of DESs decrease in the order of oxalic/glycolic acids (C_2_) > malonic acid (C_3_) > glutaric/levulinic acids (C_5_). As the length of alkyl chain increases, the corresponding molar volume increases, and the density of the DES decreases [25].

The hydrogen bonding between HBA and HBD is the main driving factor for DES formation. The increase in the number of −OH functional groups in HBD resulted in a higher number of hydrogen bonds. The higher number of hydrogen bonds reduces the free spaces available and consequently increases the density of DESs. DESs obtained from benzyl trialkylammonium chloride salts as HBAs and ethylene glycol have lower densities than DESs composed using diethylene glycol, triethylene glycol, and glycerol [37]. As the molecular weight of PEG increases from 200 to 600, the density of tetraalkylammonium bromide increases due to its stronger interaction with TBAB [33].

The density of carbohydrate-based DESs containing ChCl, acetylcholine chloride (Ac ChCl), and benzylcholine chloride (BzChCl) as three different HBA have been reported [40]. The carbohydrates used were D-fructose, D-glucose, ribose, xylose, and mannose. For common HBA, the density of DESs observed in order: (HBA):mannose < (HBA):glucose = (HBA):fructose < (HBA):ribose < (HBA):xylose. The lowest density was recorded for DESs containing xylose. A similar trend was observed for DESs composed of ethyl ammonium Chloride (EACl) with urea, 1-(trifluromethyl)urea and acetamide [44]. When *N*,*N*-Diethyl ethanolammonium chloride (DEACl) or methyltriphenylphosphonium bromide (MTPhPBr) as HBA combined with glycerol and ethyl glycol, the densities of DESs were found to decrease [43]. For common HBA ChCl, the densities of DESs composed of different HBDs at 1:2 molar ratio follow the order of urea > glycerol > ethylene glycol > p-Cresol.

The densities of DESs composed of tetraethylammonium chloride/bromide (TEACl or TEABr) salts combined with levulinic acid, ethylene glycol, and glycol in a 1:4 molar ratio showed a decrease in density with change in the type of HBD [48]. The same results have been reported for tetrapropylammonium chloride/bromide (TPACl or TPABr), tetrabutylammonium chloride/bromide (TBACl or TBABr), and tetrahexylammonium bromide (THABr) [49,50,51,52,53,54,55,56,57], as well as for other DESs composed of benzyltrimethylammonium chloride (BTACl), MTPPhBr, benzyltriphenylphosphonium chloride (BTPPhCl), and allyltriphenyl phosphonium bromide (ATPPhBr) [58,59,60,61,62,63]. In case of salt hydrate-based DESs, the density values decrease with the change in HBD. The highest and lowest density was observed with DESs composed of glycine (1.677 g cm^3^) and ethylene glycol (1.605 g cm^3^), respectively [64].

A similar trend was observed for studied carbohydrates when a type of HBA is considered. The densities of DESs decrease as ChCl:carbohydrate > Ac ChCl:carbohydrate > Bz ChCl:carbohydrate [38]. In both cases, the type and extent of hydrogen bonds and available free space were the main reasons for variation of density of DESs. When the alkyl chain lengths of DESs increased from ethyl to butyl for tetraalkylammonium salt and polyethylene glycol-based DESs, density values were found to decrease [33]. Kroon et al. have also reported the same trend for DESs composed of tetraalkylammonium chloride (TAACl) and decanoic acid [39]. The presence of different halides ions in HBA also affects the density of DESs. DESs composed of bromide salt of tetraalkylammoniums have a higher density than chloride salts [39,48,49]. When we consider the alkyl chain length of HBD from ethyl to butyl for tetraalkylammonium salts, the density of a DES composed using TEABr/Cl has the highest density due to the compact structure of the DES [49,50,51,52].

(2) Molar ratio of HBA to HBD: The molar ratio or composition of HBA:HBD can be used to manipulate the density of DESs [40,41,43,44,45,48,50]. Shafieet al. [40] presented densities of ChCl:citric acid DESs in three molar ratios. As the relative amount of ChCl increased than citric acid, the density values of DESs were decreased. On the other hand, the increasing amount of citric acid leads to increased density values for DESs. The densities of TBABr:PEG-based DESs were marginally increased with an increasing molar ratio of PEG [35]. DESs with 1:3 ratio have higher densities than DESs with a 1:2 molar ratio. The densities of TBABr:PEG 200 in 1:2 and 1:3 were 1.11077 and 1.11360 g·cm^−3^, respectively. The slight increase in density of DEACl-, MTPBr-, and THABr-based DESs for common HBD glycerol or ethylene glycol at molar ratio 1:2 [43].

(3) Temperature: The densities of DESs decrease with increases in temperature due to the thermal expansion of DESs. The density at 298.15 K of DESs formed by suitable combination of HBA and HBD is shown in Table 2. The effect of temperature on the density of DESs is depicted in Table 3. The effect of temperature on the density of DESs is expressed in terms of isobaric thermal expansion coefficients, which defines the available free volume of DESs. The isobaric thermal expansion coefficients are used to explain the compressible behavior of DESs.

The isobaric thermal expansion coefficient is obtained from experimental densities at different temperature of DESs:

*α_P_* = −*ρ*^−1^(∂*ρ*/∂*T*)*_P_* (1)

The linear decrease in the density of all DESs was observed with an increase in temperature. The reduction in density of DESs with a rise in temperature results from the availability of more free spaces between the HBA and HBD of DESs. The available space in DESs is related to changes in *α_P_* values and density with temperature. It can be observed that the values of *α_P_* of DESs are minimal compared to *α_P_* values of common solvents; however, they are quite similar to *α_P_* values of imidazolium-based ILs. The temperature showed little effect on density and *α_P;_* therefore, DESs expanded or compressed less in comparison to ILs and other organic solvents.

The variation in density with temperature was correlated using a linear equation

*ρ* = *a* + *bT* (2)

where *ρ* corresponds to density in g cm^−3^, *a* and *b* are the fitting parameters, and *T* is the temperature in K.

**Table 2:** Densities of studied DESs at 298.15 K.

HBA	HBD	Molar Ratio	Density (ρ) (g cm^3^)	References
ChCl	Urea	1:2	1.25	[42]
ChAc	Urea	1:2	1.206	[42]
ChCl	1-(trifluoromethyl)urea	1:1.5	1.324	[42]
ChCl	Glycerol	1:1	1.16	[43]
ChCl	Glycerol	1:2	1.18	[44,45]
ChCl	Ethylene glycol	1:2	1.12	[43,45]
ChCl	Ethylene glycol	1:3	1.12	[43,45]
ChCl	Oxalic acid	1:1	1.259	[25]
ChCl	Glycolic acid	1:1	1.195	[25]
ChCl	Malonic acid	1:1	1.231	[25]
ChCl	Glutaric acid	1:1	1.188	[25]
ChCl	Levulinic acid	1:2	1.138	[25]
ChCl	o-Cresol	1:3	1.07	[46]
ChCl	p-Cresol	1:2	1.0681	[47]
ChCl	Phenol	1: 3	1.092	[46]
ChCl	p-Chlorophenol	1:2	1.1988	[47]
ChCl	Glucose	2:1	1.2423	[41]
ChCl	D-Glucose	1:1	1.273	[38]
ChCl	D-Mannose	1:1	1.278	[38]
ChCl	D-Ribose	1:1	1.267	[38]
ChCl	D-Xylose	1:1	1.257	[38]
ChCl	D-Fructose	1:1	1.272	[38]
AcChCl	D-Glucose	1:1	1.252	[38]
AcChCl	D-Mannose	1:1	1.260	[38]
AcChCl	D-Ribose	1:1	1.243	[38]
AcChCl	D-Xylose	1:1	1.224	[38]
AcChCl	D-Fructose	1:1	1.239	[38]
BzChCl	D-Glucose	1:1	1.263	[38]
BzChCl	D-Mannose	1:1	1.272	[38]
BzChCl	D-Ribose	1:1	1.255	[38]
BzChCl	D-Xylose	1:1	1.254	[38]
BzChCl	D-Fructose	1:1	1.263	[38]
BTMACl	Ethylene glycol	1:3	1.1009	[37]
BTMACl	Diethylene glycol	1:3	1.1106	[37]
BTMACl	Triethylene glycol	1:3	1.1173	[37]
EACl	Urea	1:1.5	1.140	[42]
EACl	1-(trifluoromethyl) urea	1:1.5	1.273	[42]
EACl	Acetamide	1:1.5	1.041	[42]
DEACl	Glycerol	1:2	1.17	[43]
DEACl	Glycerol	1:3	1.21	[43]
DEACl	Glycerol	1:4	1.22	[43]
DEACl	Ethylene glycol	1:2	1.10	[43]
DEACl	Ethylene glycol	1:3	1.10	[43]
DEACl	Ethylene glycol	1:4	1.10	[43]
MTPhPBr	Glycerol	1:2	1.31	[43]
MTPhPBr	Glycerol	1:3	1.30	[43]
MTPhPBr	Glycerol	1:4	1.30	[43]
MTPhPBr	Ethylene glycol	1:3	1.25	[43]
MTPhPBr	Ethylene glycol	1:4	1.23	[43]
ZnCl_2_	Acetamide	1:4	1.36	[29]
ZnCl_2_	Ethylene glycol	1:4	1.45	[29]
ZnCl_2_	Hexanediol	1:3	1.38	[29]
TEACl	Levulinic acid	1:2	1.0939	[48]
TEABr	Levulinic acid	1:2	1.1736	[48]
TBACl	Levulinic acid	1:2	1.0310	[48]
TBABr	Levulinic acid	1:2	1.0972	[48]
TEACl	Levulinic acid	1:4	1.1020	[48]
TEABr	Ethylene glycol	1:4	1.1596	[49]
TEABr	Triethylene glycol	1:4	1.1468	[49]
TEABr	Levulinic acid	1:4	1.1669	[49]
TPACl	Levulinic acid	1:4	1.0759	[49]
TPABr	Triethylene glycol	1:4	1.1204	[49]
TPABr	Ethylene glycol	1:4	1.1314	[49]
TPACl	Levulinic acid	1:4	1.0484	[49]
TBABr	Ethylene glycol	1:4	1.0762	[49]
TBABr	Triethylene glycol	1:4	1.0976	[49]
TBABr	Levulinic acid	1:4	1.1061	[49]
TBABr	Ethylene glycol	1:4	1.1339	[50]
TBABr	Glycerol	1:3	1.1924	[50]
TBABr	Triethylene glycol	1:3	1.1426	[50]
TBACl	Glycerol	1:5	1.1417	[51]
TBACl	Ethylene glycol	1:3	1.0263	[51]
TBACl	Trietylene glycol	1:2	1.0043	[51]
TBACl	Ethylene glycol	1:2	0.9890	[52]
TBACl	PEG 400	1:2	1.0771	[52]
TBACl	Propanoic acid	1:2	1.1183	[52]
TBACl	Phenylacetic acid	1:2	1.0401	[52]
TBACl	Glycerol	1:4	1.1714	[53]
TBACl	Glycerol	1:4	1.1748	[53]
TBACl	Decanoic acid	1:2	0.9168	[59]
TPABr	Decanoic acid	1:2	0.8907	[39]
TOACl	Decanoic acid	1:2	0.8889	[39]
TPABr	Decanoic acid	1:2	0.9298	[39]
TBACl	Glutamic acid	10:1	0.9630	[54]
TBACl	Aspartic acid	9:1	0.9582	[54]
TBACl	Arginine	6:1	1.0042	[54]
TBACl	Serine	8:1	0.9906	[55]
TBACl	Threonine	9:1	0.9393	[55]
TBACl	Methionine	11:1	0.9393	[55]
TBABr	Ethanolamine	1:4	1.0547	[56]
TBABr	Ethyleneglycol	1:2	1.0045	[56]
TBABr	Glycerol	1:2	1.0426	[57]
BTACl	p-toulenesulfonic acid	1:1	1.1904	[58]
BTACl	Oxalic acid	1:1	1.1940	[58]
MTPPhBr	Ethylene glycol	1:4	1.393	[59]
MTPPhBr	Glycerol	1:1.75	1.233	[59]
MTPPhBr	Trifluroacetamide	1:8	1.123	[59]
BTPPhCl	Glycerol	1:16	1.2407	[60]
BTPPhCl	Triethylene glycol	1:8	1.140	[61]
BTPPhCl	Glycerol	1:16	1.2337	[62]
ATPPhBr	Glycerol	1:14	1.2630	[62]
ATPPhBr	Diethylene glycol	1:10	1.1563	[63]
ATPPhBr	Triethylene glycol	1:10	1.1555	[63]
FeCl_3_ 6H_2_O	Ethylene Glycol	2:1	1.605	[64]
FeCl_3_ 6H_2_O	Glycerol	3:1	1.637	[64]
FeCl_3_ 6H_2_O	Malonic acid	2:1	1.619	[64]
FeCl_3_ 6H_2_O	Xylitol	2:1	1.630	[64]
FeCl_3_ 6H_2_O	Serine	2:1	1.670	[64]
FeCl_3_ 6H_2_O	Alanine	2:1	1.628	[64]
FeCl_3_ 6H_2_O	Glycine	2:1	1.677	[64]

The densities of DESs at different temperatures have been reported extensively by many researchers [65,66,67,68,69,70,71,72]. Abbort et al. first reported the density of ChCl:urea and subsequently reported this for many DESs (Table 3). The density of all studied DESs decreases with increasing temperature. The amount of free space in the DES increases with increased temperature, leading to a faster movement of molecules, and a reduction in density of the DES is observed. The values of α_P_ for listed DESs are 0.5–0.7, higher than the values for organic solvents.

Table 3 represents the densities of DESs in temperature ranges between 298.15 K and 323.15 K. Pandey et al. [70] reported densities of ChCl:glycerol and ChCl:ethylene glycerol, ChCl:malonic acid from 283.15 to 363.15 K, which reveals crucial information on interactions present within these systems. Shahbaz et al. have measured the density of nine DESs composed of HBAs (ChCl, DEACl, MTPBr) and HBDs (glycerol, ethylene glycol) at various temperatures and compositions. Both have a significant effect on the densities of DESs. The group contribution and artificial intelligence methods have been applied to predict the density of a DES. The increase in glycerol mole fraction increased densities of a DES composed of ChCl and DEACl due to a higher glycerol density than that of the corresponding DES constituents. In contrast, the negative effect was observed for MTPBr-based DESs. Similarly, for DESs composed of ethylene glycol, the density of a DEACl-based DES was found to increase and decrease for ChCl-based DESs and MTPBr-based DESs [43].

**Table 3:** The density of DESs at different temperature ranges.

DESs	298.15 K	303.15 K	308.15 K	313.15 K	318.15 K	323.15 K	Ref.
ChCl:TEG	-	1.1254	-	1.1189	-	1.1125	[41]
ChCl:Glucose	-	1.2397	-	1.2346	-	1.2294	[41]
TBABr:PEG 200	1.0976	1.0942	1.0906	1.0872	1.0837	1.0802	[33]
DEAC:Glycerol	1.1766	1.1735	1.1703	1.1672	1.1642	1.1582	[65]
DEAC:Ethylene glycol	1.0999	1.0968	1.0938	1.0909	1.0879	1.0848	[65]
ChCl:Ethylene glycol	-	-	1.1109	1.1081	1.1053	1.1025	[66]
ChCl:Glycerol	-	1.1941	-	1.1913	-	1.1884	[67]
ChCl:Glycerol	1.1921	1.1892	1.1862	1.1834	1.1805	1.1777	[68]
ChCl:Urea	-	1.1942	-	1.1886	-	1.1832	[69]
ChCl:Ethylene glycol	1.1171	-	1.1114	-	1.1057	1.1001	[70]
ChCl:Ethylene glycol	-	1.1139	-	1.1081	-	1.1025	[71]
ChCl:Levulinic acid	-	1.1352	-	1.1285	-	1.1219	[71]
ChCl:Phenol	-	1.0934	-	1.0874	-	1.0815	[71]
ATPPhBr:TEG(1:4)	1.1871	1.1834	1.1797	1.1761	1.1724	1.1687	[72]
ATPPhBr:TEG(1:10)	1.1555	1.1517	1.1480	1.1442	1.1405	1.1367	[72]
ATPPhBr:TEG(1:16)	1.1425	1.1388	1.1350	1.1313	1.1275	1.1237	[72]
ChCl:Phenol(1:2)	1.0967	1.0930	1.0901	1.0873	1.0843	-	[45]
ChCl:Phenol(1:3)	1.0921	1.0890	1.0858	1.0829	1.0795	-	[45]
ChCl:Phenol(1:4)	1.0893	1.0860	1.0819	1.0803	1.0782	-	[45]
ChCl:Phenol(1:5)	1.0870	1.0838	1.0803	1.0761	1.0736	-	[45]
ChCl:Phenol(1:6)	1.0852	1.0818	1.0782	1.0745	1.0717	-	[45]

TEG: Triethylene glycol.

Nicolas et al. measured the density of ChCl-based DESs at a pressure of 101.3 kPa and in a temperature range from 293.15 K to 333.15 K. In the study, ChCl was mixed with ethylene glycol, levulinic acid, and phenol as HBD in 1:2 mole ratio HBA:HBD. It is shown that the densities of the above three DESs decrease as the temperature increases in accordance with thermal expansion of the DES. The maximum thermal stability was observed for ChCl:levulinic acid due to the presence of a significant extent of intermolecular interaction between ChCl and acid. This was the main reason for the greater density of this DES among other studied DESs [71].

As discussed above, the densities of all DESs decrease with a rise in studied temperature. The values of density greatly depend upon type of HBD. It is observed that as the number of -OH groups in the DES structure increases, the density of DESs is found to increase. A similar trend was reported when the number of aromatic groups or alkyl chains of organic acids increased. A change in density corresponded to a change in the free volume of the DESs [40]. In terms of the type of HBD, the density of studied DESs was in the order of phenol < ethylene glycol < levulinic acid. The pure components′ densities also decrease in a similar trend at a constant temperature.

The influence of water content on the density of a ChCl:urea DES was measured by Shah and Mjalli [73] at two different temperatures. The molecular dynamics simulations have been performed to analyze the intermolecular interactions in pure and aqueous solutions of DESs. The results indicated the presence of urea and Cl anion interaction at a lower melting point of ChCl:urea. The Cl anion was reported to be more hydrated in comparison to urea and choline cation. The simulation studies revealed the presence of an interaction between urea:anion, which reduces the interaction between urea:urea or choline:choline, hence decreasing the melting point of the DES. The effect of water on density was divided into three sections. The number of hydrogen bonds decreases with the addition of water. In the last section, at a higher concentration of water, an anion of HBD was hydrated over its cation. Similar observations were reported for ChCl:urea at high pressure and temperature ranges between 298.15 to 323.15 K. Yadav and Pandey [74] have also studied the aqueous solution of this DES from 298.15 to 363.15 K. The quadratic expression was presented to show decreasing behavior of density with increases in temperature. Lapeña et al. [75] have also reported densities of a DES composed of ChCl:urea at 1:2 molar ratio and aqueous mixtures (x_DES_ = 0.645) at temperature ranges between 288.15 K and 338.15 K for the DES and at a temperature range between 278.15 K to 338.15 K for the aqueous DES. The density of a hydrated DES was found to be less in comparison to pure DES due to a decrease in hydrogen bonding between HBA:HBD with the addition of water [75].

Water had an effect on a ChCl-based DES composed of oxalic acid, levulinic acid, glutaric acid, malonic acid, and glycolic acid as HBD. The DESs were exposed to an open-air environment for a month, and the amount of water taken by DESs was measured, which was found to be 14 to 20 wt%. The density of DESs saturated with water (5%) was lower than that of the dried DES samples. Therefore, it was reported that trace amounts of water affect the density of DESs. As a result, the presence of water slightly affects the density of these DESs [25].

Dai et al. [76] studied the influence of water content on the physicochemical properties and structure of various natural deep eutectic solvents (NADES). A DES composed of 1,2-propanediol:ChCl:water at 1:1:1 molar ratio was diluted using deuterium oxide. The NMR experiment was performed at 40 °C to study the structure of diluted DES. The NMR results indicated that the structure of NADES was maintained below 50% (*v*/*v*) water content. Further dilution with water caused the rupture of the DES structure, and HBA and HBD existed as an individual component in the mixture. The study showed a decrease in density of a choline chloride:phenol DES by the addition of water [77].

A temperature variation of densities of three other DESs such as ChCl:ethylene glycol, ChCl:malonic acid, and ChCl:oxalic acid was carried out by Shekaari et al. [78]. The highest density was observed for ChCl:malonic acid > ChCl:oxalic > ChCl:ethylene glycol. Kuddushi et al. [79] measured the densities of an aqueous solution of ChCl:glutaric acid and ChCl:malonic acid DESs at temperature ranges between 293.15 K and 323.15 K. The densities of both DESs decrease with temperature because of increases in thermal expansion and available free space between DES components. The density of ChCl:malonic acid DES was greater than that of ChCl:glutaric acid-based DES at all temperatures. The alkyl chain length and molar mass of HBD is greater for ChCl:glutaric acid DES than for ChCl:malonic acid. The inability to compact packing between components was the main reason for the lower density of glutaric acid-based DESs. The higher α_P_ for ChCl:glutaric acid than for ChCl:malonic acid suggests more expansion in the former. The densities of an aqueous solution of both the DESs were decreased with an increase in the amount of water and temperature.

Mjalli and Ahmad [41] reported the density of an aqueous solution of ChCl-based DESs composed of triethylene glycol and glucose at different temperatures. The comparison was presented for the measured density of aqueous solution of these DESs with the other four aqueous DES solutions reported in the literature. On comparison, it was concluded that DES densities can be ranked in the order of ChCl:glucose > ChCl:glycine > ChCl:urea > ChCl:malonic acid > ChCl:triethylene glycol > ChCl:ethylene glycol. It can be seen that DESs composed of ChCl:glucose have a higher amount of packing, whereas poor packing was observed for ethylene glycol molecules. The aqueous DES solution showed similar behavior on the addition of water. When water was introduced into pure DESs, the hydrogen bonding between components of DESs were reduced abruptly, and hence the densities of the aqueous solution of DESs decreased continuously. A similar effect of breakdown in hydrogen bonding was noticed with a rise in the temperature. This effect caused an increase in free volume in DESs, a dislocation of components of DESs, and a reduction in density of DESs.

### 3.2. Viscosity

The viscosity *η* describes the internal friction or resistance experienced by moving fluid or is defined as the resistance of moving fluid in reference to deformation at a given shear rate. The viscosity of DESs has been extensively reported due to its industrial importance. The applicability of a solvent as a reaction medium can be decided from the viscosity of the solvent. Viscosity values at different temperatures play a vital role in equipment design calculations and understanding the activation energy and mass transport phenomena [80,81,82,83]. Considerable disagreements have been observed in the viscosity of DESs due to (i) differences in the experimental method used for viscosity measurement, (ii) different DES preparation methods, and (iii) the presence of some impurities in the sample [21]. The viscosity values for common DESs were different (6.5%) when prepared by the heating and grinding method [25].

Table 4 [25,27,33,42,45,46,50,51,59,65,82,83] indicates that most of the studied DESs have higher viscosities (>100 cP), which is much higher than the viscosity of water at room temperature. The main driving forces for the high viscosity of DESs might be related to the presence of an extensive hydrogen bond interaction between components. The dense network of DESs reduces the movement of free species in the DES [36]. The other factors that relate to the high viscosity of DESs are (i) van der Waals or electrostatic interactions, (ii) the larger size of ions and small void volumes of DESs, and (iii) the available free volume [21,23,24]. The potential application of DESs as green solvents requires lower viscosity. Few DESs also have lower viscosity, less than 100 cP. For DESs formed by the mixing ChCl with 1,4-butanediol or ethylene glycol or phenol or o-cresol, etc. [45,46,82], as presented in Table 4. Four main factors that influence the viscosity of DESs include:

(1) The nature of DES components: The viscosities of DESs are mainly affected by the chemical nature of HBAs and HBDs. The viscosity of ChCl:ethyleneglycol (1:4) DES exhibits the lowest viscosity (19 cP at 293.15 K), whereas the viscosity of DES increased up to 8500 cP at 298.15 K when HBD was replaced to ZnCl_2_ [45]. Similarly, carbohydrates such as xylitol, sorbitol, or carboxylic acids such as malonic acid as HBDs exhibit high viscosities., i.e., 12,730 cP at 293.15 K for ChCl/sorbitol [81] and 1124 cP for ChCl/malonic acid at 298.15 K [82]. This can be attributed to the intermolecular hydrogen bond network. Some reports also emphasized that the molecular structure of the HBD (molecular weight and molecular size) can greatly affect the mobility of the whole system. For DESs ChCl:EG, ChCl:glycerol, and ChCl:urea, the “choline cation” diffuse slowly than the HBD. ChCl may move more slowly than malonic acid due to the formation of a dimmer chain between malonic acid molecules.

It was shown that the DESs with dicarboxylic acid are higher than those containing a monocarboxylic acid. Moreover, the addition of one –OH or –COOH group also contributed to the rise in the viscosity of DESs, probably due to an increase in hydrogen bonding. The viscosities of DESs increase in order of ChCl:oxalic acid > ChCl:gluconic acid > ChCl:malonic acid > ChCl:glycolic acid > ChCl:levulinic acid > ChCl:oxalic acid [25]. For common HBD at 303.15 K, the viscosities of DESs change with changes in HBA from ChCl, TBABr, and DEACl [50,51,59,82,83]. The viscosities of DESs also increase with increases in the molecular weights of HBA and HBD [33].

**Table 4:** Viscosity of DESs at particular temperatures.

HBA	HBD	Molar Ratio	Viscosity (cP)	Temp (K)	Ref.
ChCl	Urea	1:2	750	298.15	[82]
ChCl	Ethylene glycol	1:2	36	293.15	[82]
ChCl	Ethylene glycol	1:2	37	298.15	[82]
ChCl	Ethylene glycol	1:3	19	293.15	[45]
ChCl	Ethylene glycol	1:4	19	293.15	[45]
ChCl	1,4-butanediol	1:3	140	293.15	[45]
ChCl	1,4-butanediol	1:4	88	293.15	[45]
ChCl	Glycolic acid	1:1	394.8	303.15	[25]
ChCl	Levulinic acid	1:2	164.5	303.15	[25]
ChCl	Malonic acid	1:2	1124	303.15	[25]
ChCl	o-Cresol	1:3	77.65	303.15	[46]
ChCl	Phenol	1:2	64.41	303.15	[46]
ChCl	Phenol	1:3	35.17	303.15	[46]
ChCl	Phenol	1:4	25.20	303.15	[46]
ChCl	Phenol	1:5	19.75	303.15	[46]
ChCl	Phenol	1:6	16.82	303.15	[46]
ChCl	D-Sorbitol	1:1	12730	303.15	[81]
ChCl	Xylitol	1:1	5230	303.15	[81]
ChCl	ZnCl_2_	1:2	8500	298.15	[27]
ChCl	Urea	1:2	750	298.15	[82]
ChCl	Urea	1:2	449	303.15	[42]
ChCl	Urea	1:2	169	313.15	[42]
ChCl	Glycerol	1:2	376	293.15	[45]
ChCl	Glycerol	1:2	259	298.15	[82]
ChCl	Glycerol	1:2	246.79	303.15	[45]
ChCl	Glycerol	1:3	450	293.15	[45]
ChCl	Glycerol	1:4	503	293.15	[45]
TBACl	Ethylene glycol	1:3	56.9	303.15	[51]
TBACl	Glycerol	1:4	476.1	303.15	[51]
TPABr	Ethylene glycol	1:3	58.2	303.15	[50]
TPABr	Triethylene glycol	1:3	71.9	303.15	[50]
TPABr	Ethylene glycol	1:3	77	303.15	[83]
TPABr	Glycerol	1:3	467.2	303.15	[83]
TPABr	1,3-propanediol	1:3	135	303.15	[83]
TPABr	1,5-propanediol	1:3	183	303.15	[83]
TBABr	Glycerol	1:3	467.2	303.15	[51]
TBABr	Glycerol	1:4	476.1	303.15	[51]
TBABr	PEG 200	1:3	115.82	298.15	[33]
TBABr	PEG 400	1:3	157.14	298.15	[33]
TBABr	PEG 600	1:3	182.32	298.15	[33]
MTPBr	Ethylene glycol	1:4	109.8	298.15	[59]
DEACl	Glycerol	1:2	351	303.15	[65]
BTMACl	Glycerol	1:5	553.7	328.15	[59]
EtNH_3_Cl	Acetamide	1:1.5	64	313.15	[42]
EtNH_3_Cl	CF_3_CONH_2_	1:1.5	256	313.15	[42]
EtNH_3_Cl	Urea	1:1.5	128	313.15	[42]

(2) Molar ratio of HBA:HBD: In general, higher molar ratios of HBA to HBD resulted in higher viscosities of DESs. The amount of free volume decreases due to the compact structuring of DESs. The small amount of free volume leads to slow molecular motion and higher viscosity of DESs [2].

The viscosities of DESs decrease with increases in the molar ratio of HBD. The viscosity of a DES increases with an increase in the molar ratio of glycerol for ChCl/glycerol DES. The viscosity values of DESs at 1:2, 1:3, and 1:4 molar ratios were reported around 503, 450, and 376 cP, respectively, at 293.15 K. The addition of ChCl to glycerol leads to a breakdown of hydrogen bonding and decreased viscosity of DES [44]. A similar trend was observed with ChCl:1,4-butanediol DES [45] and TBABr:PEG [33]. In some DES ChCl:ethylene glycol [45], no change in the viscosity was observed between 1:3 and 1:4 (19 cP at 293.15 K), as shown in Table 4. However, the reverse trend was observed in the case of ChCl:phenol DES, where viscosity decreased with an increase in the molar ratio of phenol [46].

(3) Water content of DESs: Water content in DES is one of the most important criteria when considering the viscosity of DES. Since most DESs are highly hygroscopic, it is essential to provide the water content of DESs. The viscosity of DES composed of ChCl:urea (1:2) decreased to 200.6 cP from 527.3 cP in the presence of 0.1 mole fraction of water [74]. Depending upon HBDs, the viscosity of DESs decreased 10 to 30 times in the presence of water. In ChCl:oxalic acid (1:1 molar ratio), viscosity was reduced around 44 cP from 5363 cP when it absorbed 19.40% of moisture. The viscosity of DES can be tuned by adding a known amount of water [25].

(4) Temperature: As per general observation, the viscosity of DESs decreases with a rise in temperature; therefore, DESs with high viscosity at ambient temperature can be used at a higher temperature. The viscosity of ChCl:urea (1:2), ChCl:glycerol (1:2), ChCl:malonic acid, and ChCl:glucose decreases from 750 to 95 mPa s, 259 to 52 mPa s, and 1124 to 161 mPa s and 7992 mPa s to 262 mPa s between temperature ranges between 298 and 328 K [45,82].

The temperature-dependent viscosity behavior of DES was explained using Hole theory. The application of the Hole theory for the viscosity of DES was studied by Abbott and co-workers [35,42,45]. According to this theory, the viscosity and electrical conductivity depend upon the presence of holes in the liquids that facilitate the mobility of ionic compound in network. The viscosity is said to control by volumetric factors rather than stronger interactions between HBA HBD. This theory quantified the contribution of the steric effect in addition to interactions on viscosity of DES. The distribution of holes defined as the probability of the presence of a hole in radius in choline and tetrabutylammonium bromide-based DES of phenol, glycerol, ethylene glycol, and malonic acid was reported [23]. It was reported that the distribution of the size of holes depends upon the type of both HBA and HBD. Hole theory assumes that ionic material contains empty spaces on melting, which arise due to a change in the density of liquids with temperature. The holes are at different locations and sizes and in continuous motion. As per Hole theory, at a lower temperatures, the sizes of holes are small compared to the size of components of DES; hence, the latter are difficult to fit into the holes, reducing the free mobility of components. This is why DES systems have much higher viscosity for 100–1000 Pa at a lower temperature. The average size of holes becomes comparable to the size of components as temperature increases. The small components can easily move to holes and increase the mobility of DES. It was assumed that the cavity in DES moved in the opposite direction to the solvent molecules. At a given temperature, a component of DES can only move if there is an availability of a cavity of similar size.

The two most commonly used models applied to DESs to study the effect of temperature on viscosity are (1) the Arrhenius model and (2) the Vogel–Fulcher–Tamman (VFT) model [21,24,37,74]. The Arrhenius equation gives a correlation between the viscosity and temperature of DES.

(3) $$\ln\eta =\ln\eta_{\infty }+\frac{E_{\eta }}{RT}$$

where *η*_∞_ is a pre-exponential constant, ln*η* is logarithm of viscosity, *T* is temperature, *R* is gas constant, and $E_{\eta }$ is the activation energy of viscosity.

The relation of DES viscosity with a strength of intermolecular interaction and temperature can be obtained by fitting viscosity at various temperatures in this equation. The activation energy (*E_a_*) can be obtained using the Arrhenius equation. Less viscous DESs such as ChCl:ethylene glycol show very low *E_a_* values, whereas highly viscous DESs such as ChCl:glucose have larger *E_a_* [21]. In the case of ChCl DES with oxalic acid, malonic acid, and glutaric acid, the calculated *E_a_* were −65.2, −46.7 and −47.6 kJ mol^−1^, respectively [23]. The DES with extensive hydrogen bond showed higher values of *E_a_*; therefore, HBA:HBD interactions are crucial for viscosity of DES. For molten salts, it was reported that the activation energy for viscosity is linearly related to melting temperature (*E_a_* = 3.7 RTm) [21]. A similar trend was reported by Abbott et al. [36] for choline and tetraalkylammonium-based DES.

Another approach to correlate the viscosity of DES with temperature is the Vogel–Fulcher–Tammann (VFT) model [74,80].

(4) $$\ln\eta =A+\frac{B}{\left(T-T_{0}\right)}$$

ln*η* is logarithm of viscosity, and *A*, *B*, and *T*_0_ are fitting parameters. *T*_0_ is the Vogel temperature, where all atoms′ movement is considered frozen.

The viscosity of ChCl-based DESs composed of p-toluenesulfonic acid, monochloroacetic acid, trichloroacetic acid, and propionic acid as HBDs showed better fitting using the VFT model than the Arrhenius [35]. Several natural DESs composed of betaine or ChCl showed Newtonian behavior with sugar molecules and were modelled using the Arrhenius equation [35]. A similar study was conducted for the viscosity of benzyltrialkylammonium-based DESs and found that the VFT equation gave better fitting for results than the Arrhenius equation [37]. Mjalli et al. have developed two new empirical models to correlate temperature and composition-dependent viscosity behavior using Eyring and VFT models. The models were verified for ChCl-based DESs’ accuracy. The results showed that the Eyring-based viscosity model was more accurate for low-viscosity DESs, whereas for higher viscosity DESs, the VFT model better matched experimental viscosities [55]. The equation used to correlate viscosity by Eyring-based viscosity model is shown as:

(5) $$\eta =\frac{hN_{A}}{V_{m}}\exp\frac{E_{0}}{RT}$$

where *N_A_*, *h*, and *R* indicate Avogadro’s number, Planck′s constant, and ideal gas constant, respectively. *V_m_* is molar volume, *η* is dynamic viscosity of liquid, and *E*_0_ is activation free energy for viscous flow.

## 4. Applications of DES

### 4.1. Drug Delivery/Solubilization

The drug discovery and drug delivery of new drugs often face challenges such as safety, efficacy, cost, and availability [84,85,86,87,88]. To overcome this, existing drugs are now modified to improve their formulation or drug conversion (salts/ester), to introduce new combinations of existing drugs, or to change the route of drug administration [84,85]. The transport and processing of pharmaceuticals requires a solvent, and this purpose is fulfilled using water, unless the drug is a hydrophobic drug that is poorly soluble or insoluble [85]. Due to low permeation and bioavailability, the low solubility of available or developing drugs influences the therapeutic action [85]. For the oral drug delivery system, the improvement of drug bioavailability and solubility limits the drug delivery/administration [85]. One of the strategies is to have an improved formulation, with active pharmaceutical ingredient (API) being encapsulated, dispersed, or loaded inside a drug carrier [85,86]. One such study is the absorption of sulfathiazole from a eutectic mixture with urea compared to the absorption of ordinary sulfathiazole [87]. Thus, various studies [81,82,83] are conducted on deep eutectic solvent (DES) and eutectic mixture to improve the API solubility and dissolution behavior.

The approved drugs or drugs under development are poorly water-soluble, so one of the characteristics to improve the drug efficiency, permeability, and bioavailability is enhancing drug hydrophilicity [85,88]. For instance, manipulating drug formulation may increase the solubility and dissolution rate of BCS class II (Biopharmaceutics Classification System II) substances in gastrointestinal fluids, increasing bioavailability [85,89]. The efficiency of poorly water-soluble drugs can be improved by modifying API dosage, novel drug administration routes, and adopting a suitable combination of active ingredients [85,88]. One strategy is to form API dispersion inside a biocompatible polymer matrix or search for alternative solvents [81,84]. In this regard, ionic liquids have been utilized as a suitable solvent system for API, owing to the unique physicochemical properties of IL [85,86,87,88]. A suitable cation–anion combination can be made to synthesize numerous ILs [85] with appropriate physical properties desirable for the dissolution/loading of APIs [85]. However, ILs still suffer from biodegradability or toxicity limitations [85]. So, a search for a new biocompatible solvent system with negligible toxicity for API dissolution or solubility is required to improve and develop drug formulations [85,88].

One such biocompatible, cheap, less toxic solvent being studied is based on DESs [85,88,89]. DESs have been used for the dissolution of API, and DESs are prepared using pharmaceuticals as one of the components. So, the following section describes these two applications of DESs.

### 4.2. Therapeutic Deep Eutectic Solvents (THEDES)

DESs have promising properties required for successful drug formulations. The choice of components for DES preparation provides an opportunity to tailor the solvent system to various applications, including drug formulation, delivery, or dissolution [76,90,91]. DESs show an enhanced solubilizing power for drug molecules; for instance, the itraconazole solubility was 22,000–53,000 times higher in choline-based DES than the solubility of the drug in water [90,91,92,93]. The formulation with higher drug concentration leads to apparent drug supersaturation when diluted in gastro-intestinal fluid and can cause risk due to drug precipitation [90]. Thus, DES-based drug delivery is termed a supersaturating system [90,93]. Upon drug precipitation, the free drug available will reduce and hinder forces responsible for drug permeation [90]. Precipitation inhibitors are used in the formulation [90,94,95] to mitigate the precipitation effect.

Enhancing drug solubility and modulating drug release kinetics are two strategies to overcome problems associated with drug molecules′ low bioavailability/low solubility [96]. Enhanced skin permeation was, for DES, composed of ibuprofen and terpenes [97]. Ibuprofen was combined with several terpenes (menthol, thymol, 1,8-cineole, menthone, d-limonene, and p-cymene). The transdermal penetration experiments were performed to evaluate the DES-based system′s effectiveness for drug solubility, absorption, and permeation [97,98]. The composition fine-tuning can form DES with one of the components as API, and this can be termed as a therapeutic deep eutectic system (THEDES) [98]. When administered in the form of DES, API′s solubility, intestinal absorption, controlled release, and efficient transport across membrane has been improved significantly [96,97,98,99].

APIs such as ibuprofen, phenylacetic acid, and acetylsalicylic acid were combined with commonly used HBAs (choline chloride or menthol) to form THEDES. These eutectic solvents can have melting points above or below ambient temperature [96]. The eutectic solvents bearing API as one of the components are shown in Table 5 [97,98,99,100,101,102,103,104,105].

**Table 5:** The HBAs and HBDs used for the preparation of THEDES.

HBD	HBA	Molar Ratio	Ref.
DL-menthol	Ibuprofen	2.5:7.5	[97]
L-menthol	Ibuprofen	3:7	[97]
Methyl nicotinate	Ibuprofen	1:1	[97]
1,8-Cineole	Ibuprofen	2:3	[97]
Lidocaine	Ibuprofen	1:1	[97]
Decanoic acid	Propranolol	3.5:6.5	[100]
Phenol	Itraconazole	2.4:7.6	[101]
Glycerol	Ranitidine	2:1	[102]
Aspirin	ChCl	1:1	[103]
Phenylacetic acid	ChCl	3:2	[103]
Ascorbic acid	ChCl	2:1	[104]
Salicylic acid	ChCl	1:1	[105]
Paracetamol	ChCl	1:1	[105]
Thymol	ChCl	1:2	[105]

Skin is the largest organ of the body, which acts as a protective barrier against the external environment and absorbs exogenous molecules [106]. This skin barrier (due to *stratum corneum*) makes penetration of exogenous molecules challenging [106]. Thus, topical drug delivery systems require appropriate modification and technology to cross this barrier [106] or to weaken the *stratum corneum* using various invasive and non-invasive techniques [106,107,108]. Among these, absorption promoters or permeation enhancers are used. The former increases the cutaneous permeability by modulating the thermodynamic action or the partition coefficient of the API or by influencing the lipid structure, thus changing the composition of the *stratum corneum* [106]. Nonetheless, the absorption promoter or permeation enhancers should be non-toxic, biocompatible, drug compatible, free from adverse pharmacological activity, and cheap [106,109]. In this regard, DESs and ILs have been evaluated [109,110,111,112]. Ionic liquids (ILs) with low vapor pressure and various promising physicochemical properties have also been used as an alternative solvent to organic (volatile) solvents [113,114].

**Figure 4:**
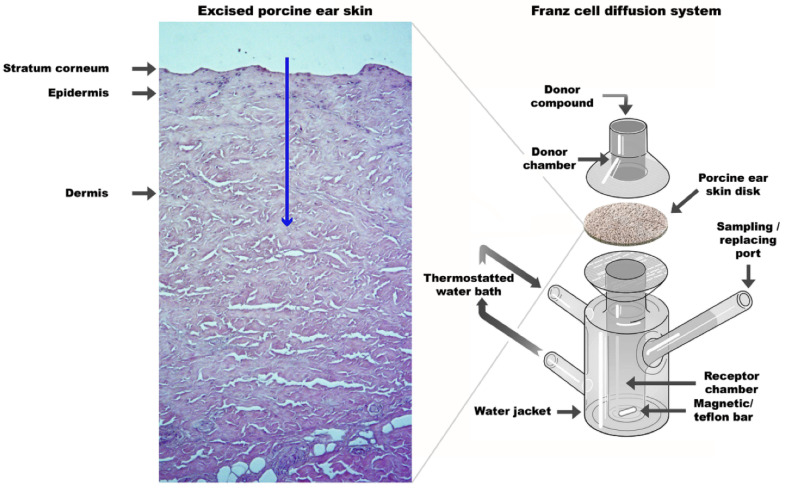
Diagram showing static Franz diffusion cell for permeation tests (in vitro) of DES-based formulation, blue colored arrow shows drug permeation pathway [106]. Reproduced from ref. [106] under terms of CC BY 4.0 license.

Boscariol et al. [106] have recently used a DES-based formulation to study the enhanced transdermal delivery of bioactive compound (curcumin) across model porcine ear skin (skin excised from pig ear). Choline bicarbonate:geranic acid (1:2) was used to prepare DES, and in vitro skin permeation tests were performed using suspension containing curcumin as a bioactive compound with DES [106]. The Franz diffusion cell, as shown in Figure 4, was used to evaluate the in vitro drug permeation [106], and the pathway for drug permeation is depicted.

A virtually nil genotoxicity effect of DES on 3T3 cell was observed, which indicated a lack of cytotoxicity of DES [106]. As shown in Figure 4, DES with various compositions in contact with porcine ear skin showed no significant changes to the skin [106]. Possible deconstruction in the *stratum corneum* was observed for a few concentrations of DES in contact with skin. The DES-based formulation with a DES composition of 2% showed a maximum average permeated curcumin concentration of 375 ng_curcumin_/mm^2^ after 15 min of transdermal application to modelled skin. The results showed that DES promoted transdermal permeation of the bioactive curcumin molecules. Thus, DES can be employed for topical formulations for enhanced delivery by a non-invasive technique to deliver various poorly water-soluble bioactive molecules [106]. For transdermal delivery of curcumin, various drug delivery systems were employed [115,116,117,118]. These delivery systems were checked for drug permeation using Franz diffusion cell, utilizing animal skins (rat, pig) [114,115,116,117]. The drug delivery systems were based on microemulsion, nanocrystals, and smart films [115,116,117,118,119]. The drug permeation was studied over periods of time ranging from minutes to hours [115,116,117,118,119]. The permeation enhancers or lipophilicities were also used for enhanced drug permeation. A detailed comparison is thus avoided.

For DES formed from choline chloride with API, various interactions can occur, such as alkyl–alky interactions, cation–pi, anion–pi, halogen bonds, and hydrogen bonds [96,97]. Among these non-bonding interactions, hydrogen bonding plays a crucial role [96,97]. Yin et al. [119] fabricated a new drug delivery system based on DES with one of the ingredients as a natural product derived from APIs, namely paeonol and osthole. Paeonol, a phenolic compound present in traditional Chinese medicine, and osthole, a coumarin derivative extracted from *Cnidium monieri (L.) Cusson* fruits, have shown various pharmacological functions such as anti-bacterial, anti-inflammatory, anti-oxidant, and anti-cancer properties [119,120,121,122]. These APIs′ applications are hindered due to the low solubility of 6 μg/mL of osthole and 0.54 mg/mL of paeonol [119].

THEDES and microemulsion gel were made using these APIs and evaluated for their transdermal delivery performance [119]. The THEDES have shown enhanced water solubility of APIs and a better permeation behavior. The authors indicated from their results that THEDES might serve the purpose of a better drug delivery system with the simplicity and convenience of constructing a multifunctional drug delivery system that can be more applicable for transdermal drug delivery [119,120].

**Scheme 1:**
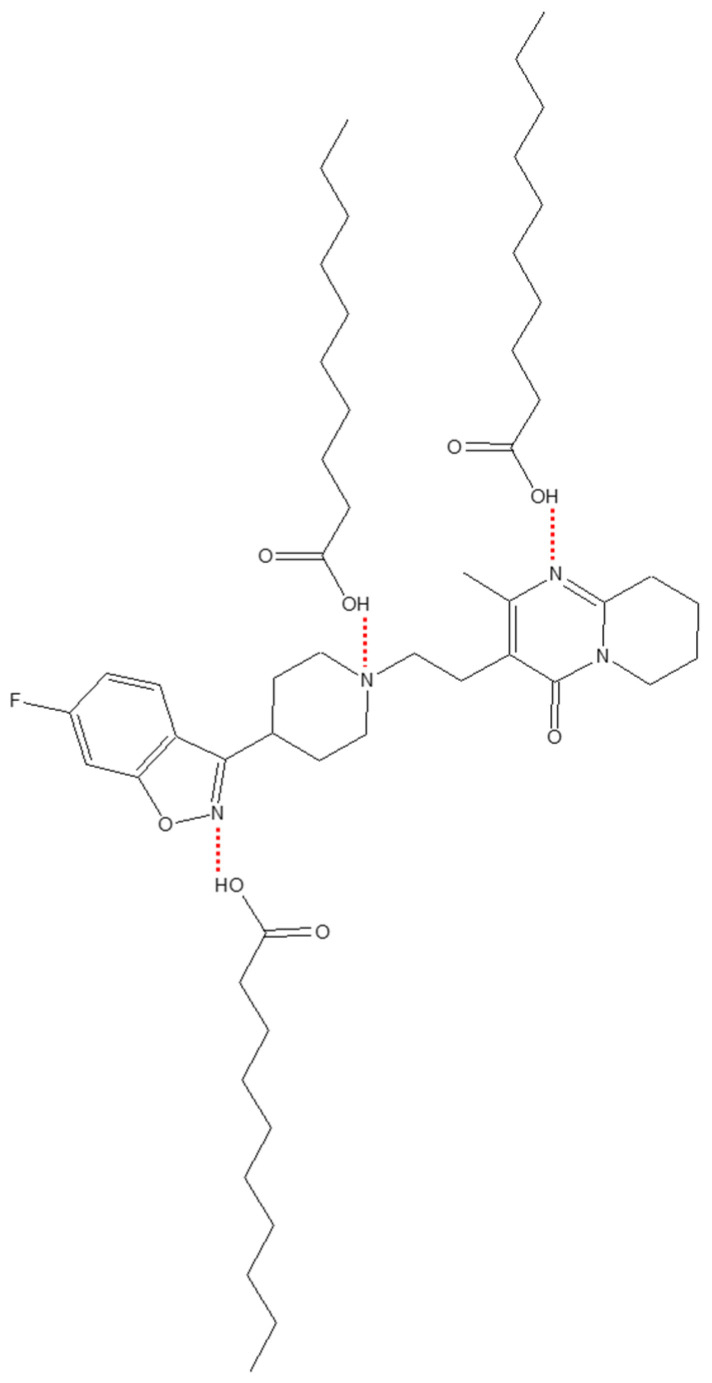
The mechanism of interaction between capric acid and risperidone [123].

A fatty acid containing THEDES was formulated using the antipsychotic drug risperidone [122]. The benzisoxazole derivative is used for the treatment of bipolar disorder, irritability in adolescents, and children with autism [122]. The drug has poor solubility in water and ethanol [122] and suffers from low bioavailability due to its drug-protein binding and hepatic first-pass metabolism [122,123]. The liquid formulation (eutectic mixture) demonstrated stability with no crystal formation at room temperature. A possible mode of interaction between the fatty acid and drug is shown in Scheme 1.

The stable viscous mixture was obtained by gently mixing components without temperature treatment, mechano-chemical agitation, or solvent [123]. The formulation was evaluated for its drug permeation abilities on rat skin [123]. The permeation was performed by selecting THEDES with a melting point below the temperature of permeation studies (32 °C). The natural fatty acid containing THEDES and the low melting mixture enhanced drug penetration [123]. The formulation on shaved skin of rats showed no edema effects or any effect of skin irritation [123]. The highest drug flux of 1509.9 ± 98 μg/cm^2^ h was achieved for the formulation having the lowest melting point of 17 °C, and the drug permeation studies reveal that high drug accumulation in *stratum corneum* (hydrophobic part of the skin) may be due to the hydrophobicity of the eutectic mixture containing the drug risperidone [123].

Recently, the first report of a clinical translation of choline-based DES for the treatment of rosacea was presented [124]. The result discussed the therapeutic potential of choline geranate DES/ionic liquid (CAGE) and the potential use of CAGE for dermatological applications [124]. The authors reported the large-scale synthesis of therapeutic CAGE in a commercial reactor, and the synthesized CAGE was characterized using TGA, modulated differential scanning calorimetry (MDSC), and FTIR. The CAGE was used as an antimicrobial agent for treating rosacea, an inflammatory disorder that influences the skin (face). Therapeutic action was evaluated by treating the root cause of rosacea, which, as per the literature [125,126], may be because of the kallikrein five enzyme activity and due to the role of resident bacteria [125,126]. The effective antibacterial and enzyme inhibition concentrations of CAGE were low, i.e., <0.45 mM and 30.84 mM, respectively [124]. The biocompatibility of CAGE was evaluated on pigs and humans. It demonstrated the therapeutic potential of the DES-based formulation with a reduced number of inflammatory lesions in the cosmetic study [124].

THEDES based delivery systems have also been studied for tuberculosis therapy by incorporation of L-arginine forming THEDES into a polymer matrix using supercritical CO_2_ technology [127]. Citric acid:L-arginine:H_2_O compositions were optimized for encapsulation in glycerol monostearate particles. The methodology was adopted to administer powdered inhalable particles for fast and localized delivery of the drug [127]. Phase equilibria results on THEDES and CO_2_ phases were used for selecting particles from gas saturated solution (PGSS) for THEDES encapsulation [127].

**Figure 5:**
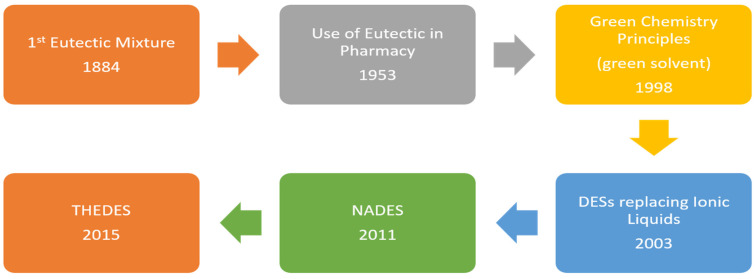
Time evolution of DES since their reference (eutectic solvent) to its promising properties according to principles of green chemistry to the replacement of ILS and incorporation of API forming THEDES [128].

DESs have been employed as vehicles for the solubilization of pharmaceutical ingredients. DESs have also been prepared with one of the components as the active pharmaceutical ingredients API (known as THEDES) [128]. This green chemistry approach solves the problems associated with the bioavailability and toxicity of APIs [127,128]. The cost of production, safe products, non-toxic properties, and reduced risk of contamination are some of the factors which govern the sustainable approach for having a therapeutic agent [128]. Among the active areas of green chemistry, one solution is to have a biocompatible green solvent system, as presently used solvents can be corrosive, toxic, and contribute towards waste generation [128,129]. So, a process can be designed that either avoids using harsh solvents or uses alternative benign solvents (e.g., water, DES). DES or THEDES provides an opportunity to be used as a non-invasive biocompatible formulation used to treat various ailments ranging from skin disease to tuberculosis [98,124,125,126,127,128,129]. THEDES can be prepared with diverse combinations of active components, allowing the modulation of properties, features, and applications in pharmaceuticals. The evolution of DES for pharmaceutical applications with the timeline is presented in Figure 5. The concept of green chemistry in pharmaceuticals is highly desirable for therapeutic formulations that are cost-effective, less or non-toxic, ecological alternatives to current pharmaceuticals, and that improve the drug properties [128].

### 4.3. Extraction of Biomolecules

According to the principle of green chemistry, solvents or auxiliaries can be avoided; if not, they should be non-toxic or harmless [130]. Green solvents must possess health, environmental, and safety characteristics not found in conventional solvents [130,131,132]. Solvents are often used in surplus amounts and have been categorized by their solubilization ability, flammability, toxicity, and volatility [130,132]. Solvents have been used extensively in synthesis, separation, and extraction [130,131]. These limitations were minimized; alternative solvents such as ILs and DESs have been synthesized/prepared, characterized, and studied for their physicochemical properties [113,114,130,131,132]. This search for non-toxic, environmentally compatible solvents also leads to exploring the sustainable chemical process, thus prioritizing green analytical chemistry and green chemistry [130].

The development of novel analytical procedures requires miniaturizing the system and improving selectivity and sensitivity [131]. One of the crucial roles in analytical chemistry is sample preparation, whereby the determination of trace amounts of compounds, analyte extraction, or extraction of a class of compounds in a given solvent is performed for further analysis [131,132,133]. Conventional sample preparation or analysis techniques can include multi-step solid–liquid extraction or liquid–liquid extraction, which can be time consuming or laborious with the use of large solvent volumes [133]. The production of waste from these extraction processes can be harmful to humans and the environment [133]. Recently, researchers have focused on finding a novel solvent system or on developing processes that do not require any solvents [128]. For instance, IL and DES are considered potential candidates with large solubilization window, low volatility, low toxicity, and ease of preparation [132,133]. DESs are easy to prepare, as one of the components can be a natural component (forming natural deep eutectic solvents, NADES) [132,133]. Appropriate mixtures containing natural products (sugars, amino acids, organic acids, etc.) can show a similar behavior to that of DESs [134,135].

DESs have demonstrated excellent ability to be used in the extraction process [134,135]. As discussed above, DESs have shown promising results when used as a solvent system for the solubilization of drugs [134,135,136,137]. NADES can be hydrophilic or hydrophobic, and the former involves preparation using natural plant metabolites such as sugars, amino acids, and organic acids. At the same time, the latter requires a combination of menthol with acetic, pyruvic, lauric, or lactic acid, thymol-coumarin and -menthol, borneol with oleic, or decanoic acid [135,136]. Hydrophilic or hydrophobic NADES can be prepared by suitably combining HBDs and HBAs.

In Table 6 are shown various types of bioactive compounds along with their sources, which are extracted using DESs, as mentioned [137,138,139,140,141,142,143,144,145,146,147,148,149,150,151,152].

**Table 6:** The bioactive compounds that were extracted from various plant sources using DESs.

Natural Source	Bioactive Compounds	Ref.
Chinese rhubarb (*Rheum palmatum* L.)	Chrysophanol, physcion, rhein, emodin, aloe-emodin	[137]
Red Sage (*Salvia miltiorrhiza*)	Tanshonene IIA, cryptotanshinone, salvianolic acid B	[138]
Grape pomace (*Vitis vinifera*)	Anthocyanin	[139]
Mulberry leaves (*Morus alba* L.)	Gallic acid, vanillic acid, benzoic acid, gentisic acid	[140]
Mulberry leaves (*Morus alba* L.)	Quercetin, Astragalin, rutin, syringic acid, chlorogenic acid, catechinic acid	[141]
Marjoram (*Origanum majorana*), Mint (*Mentha spicata*), Sage (*Salvia officinalis*), Fennel (*Foeniculum vulgare*), Dittany(*Origanum Dictamnus*)	Total polyphenol	[140]
Olive pomace (*Olea europaea*)	Total phenol	[141]
Grape skin (*Vitis vinifera*)	Anthocyanin, total phenol	[143]
Cinnamon bark (*Cinnamomum burmanniiis*)	Coumarin, trans-cinnamaldehyde	[144]
Corn mint (*Mentha arvensis*)	Total flavonoid, total phenol	[145]
Rosemary (*Rosmarinus officinalis* L.), Chinese hickory peels (*Carya cathayensis* Sarg.), Mudou leaves (*Cajanus cajan*), French lavender (*Lavandula pedunculata*)	Total phenol	[146,147,148,149]
Roselle (*Hibiscus sabdariffa* L.), Alkanet root (*Alkanna tinctoria*), Chickpea (*Cicer arietinum* L.) sprouts	Total phenol, total flavonoid	[150,151,152]

Improving the extraction process is needed to recover the maximum content of bioactive compounds; in this regard, various advanced technologies have been employed [135,136]. Ultrasound-assisted extraction (UAE), pressurized-liquid extraction (PLE), microwave-assisted extraction (MAE), ultrasound–microwave-assisted extraction (UMAE), homogenate-assisted extraction (HAE), and pulse–ultrasound-assisted extraction (PUAE) are some of the extraction techniques employed for improving the extraction process using NADES as a solvent system [136]. A bottleneck for conventional extraction is a time-consuming, laborious process; for instance, the valuable phenolic and flavonoids have been extracted from olive leaves [153]. Conventionally, these important compounds from olive leaves have been extracted using the solvents hexane, methanol, ethanol, and dimethyl sulfoxide [153]. The extraction rate can be enhanced by high-temperature treatment, solvent system change, or mechanical agitation [153]. However, high-temperature treatment reduces the extracted content of phenolic compounds, so non-conventional techniques were employed [153]. These include microwave, ultrasound, pressurized-liquid extraction, variable electric field or voltage applications [153]. Among these, which requires less energy input, as in the case of ultrasound-assisted extraction, is considered a green process [153]. Moreover, green solvent alternatives to petroleum-derived harsh chemicals have been explored as promising candidates for extraction [153]. NADES were used for the extraction of chemicals from olive and grape pomaces [153], cinnamon, sesame, olive oil [153,154,155], and agricultural waste [153,154,155,156]. The combined use of ultrasound and NADES has also been employed to extract biomolecules [153,154,155,156].

Recently, the first NADES based on a glucose–fructose–water mixture was used for the extraction of bioactive compounds assisted by ultrasound and was reported in caffeic acid (112.77 mg kg^−1^ dw) in large amounts [153] and oleuropein (1630.80 mg kg^−1^ dw) [153] from olive leaves when NADES was used instead of methanol (extraction of caffeic acid was 41.54 mg kg^−1^ dw and oleuropein was 1221.17 mg kg^−1^ dw) [153]. Among the various bioactive compounds, phenolic compounds (hydroxysaffor yellow A, oleacin, α-mangostin, oleocanthal, genistein, caffeic acid, and chlorogenic acid) have been extracted from various biological sources [153].

#### 4.3.1. Flavonoids

**Figure 6:**
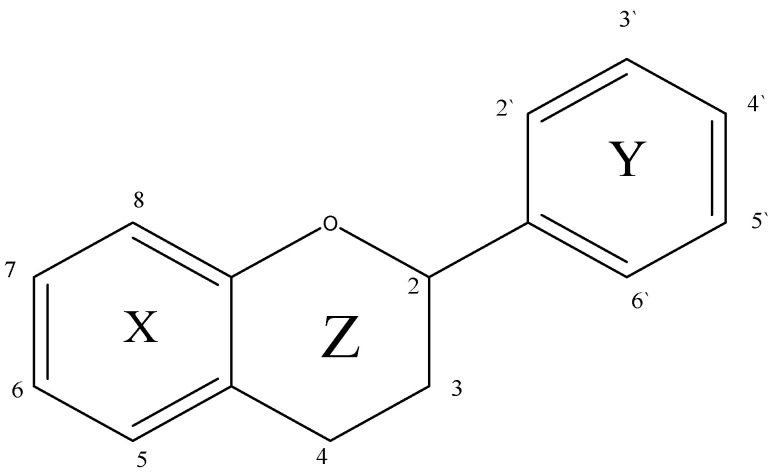
The basic flavonoid structure.

**Figure 7:**
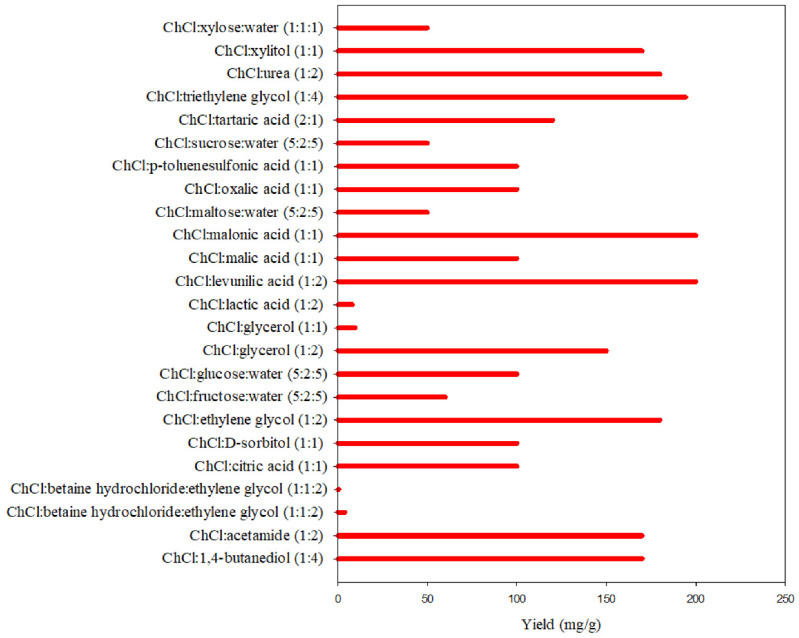
The extraction of various flavonoids (wogonoside, luteolin-7-*O*-β-d-glucopyranoside and rutin) using DES along with extraction yield. The data point not highlighted refers to rutin’s extraction and extraction yield (from Ref. [164]). The Refs. [164,165,166] provide the mode of extraction and extraction conditions.

The class of natural compounds with various phenolic structures with anti-cancer, anti-inflammatory, and anti-oxidant properties [157,158,159,160] are also extracted from natural sources [157]. Various flavonoid compounds have been extracted using DES. The phytochemical present in legumes, roots, leaves, fruits, vegetables, and cereals has various medicinal and nutritional properties [160]. Besides protection from UV light, these secondary metabolites prevent the oxidation of fat and protect enzymes and vitamins in plants [160]. Owing to these properties, humankind has been fascinated by flavonoids [160]. Thus, various extraction methods have been utilized to extract these compounds. The low molecular weight flavonoids with flavone skeleton having phenyl rings (X and Y) linked via a heterocyclic pyrene ring Z bearing an oxygen atom and the flavonoid with a typical 15 carbon skeleton (C6-C3-C6) are shown below in Figure 6 [160,161]:

The carbon atoms are numbered in the rings X and Z as 2–8, and those in ring Y are numbered 2′ to 6′ [160,161]. Many organic solvents have been used to extract flavonoids by employing techniques such as percolation, maceration, boiling, hydro-distillation, Soxhlet, soaking, and reflux [160,161]. To overcome limitations related to toxicity, low extraction yield, time-consuming extraction, thermal degradation, or low selectivity, advanced techniques have been used [161,162]. Among these, ultrasound-assisted extraction is based on the acoustic cavitation process, thereby forming gas bubbles due to the passage of ultrasonic waves in the liquid medium [157,160,161]. During compression rarefaction cycles of the ultrasonic waves, the bubbles collapse, causing an increase in pressure and temperature [160,161]. Under such conditions, the bioactive compounds were released due to breaking the biomaterial (e.g., plant material) [160,161]. Breaking of bubbles releases energy, causing weakening or breaking of intermolecular interactions between the biomaterial matrix and the compound of interest (flavonoids as shown in Figure 7) [160,161]. Such energy release also causes mechanical effects such as particle size reduction and cell damage, enhancing the solvent–sample interactions, leading to a better mass transfer, i.e., higher extraction yield in a short period [160,161,162,163]. The various flavonoids extracted from natural sources using DES are shown in Figure 7 and extraction yield [164,165,166].

Generally, the extraction of bioactive molecules using eutectic systems depends on various factors such as type of DES, moisture, and energy input [167]. The extraction solvent as DES and extraction conditions are thus considered for optimizing the maximum extraction yield of bioactive compounds [157,167]. Due to hydrogen bonding, DES are highly viscous, which can be modulated by high-temperature treatment or by the addition of water [167]. Thus, decreasing viscosity can enhance the extraction and penetration of targeted compounds into the solvent system, which is otherwise limited in highly viscous DES [167]. Additional water reduces the viscosity and influences the other physicochemical properties of DESs but can also influence the bioactive molecules–DES interactions (hydrogen bonding) [167]. Mechanical agitation during extraction has both advantages and disadvantages. On the one hand, the extraction yield increases but influences the repeatability of the process [167]. This mechanical agitation is provided by various techniques such as shaking, stirring, vortexing, heating, or a combination of these [167]. To overcome this supplementary energy provided from ultrasound/microwave, the extraction time has been shortened with increases in extraction yield. Once the substance is removed from the plant source, the active compound is recovered from DES using microporous resins, antisolvents, and liquid–liquid extraction [167,168,169,170].

#### 4.3.2. Polysaccharides

Polysaccharides have shown various medicinal effects such as antioxidant, anti-diabetic, antiglycation, anti-viral, anti-inflammatory, anti-coagulant, immunostimulatory, and anti-tumor properties [157,171]. Due to strong inter- and intra-molecular hydrogen bonding, the extraction of polysaccharides requires harsh treatment conditions or processes [113,114,157]. The polysaccharides from plant sources can be extracted by several methods such as alcohol precipitation, acid–base treatment, enzymatic treatment, microwave or ultrasound-assisted extraction, or extraction using alkali [157,172,173,174,175]. Water or aqueous alkali have been used for extraction, but they suffer from extraction yield for poorly water-soluble polysaccharides [173]. The presence of alkali in extraction can influence the activity as well as the structure of polysaccharides [173]. Again, to overcome the limitation of the extraction process or solvent system, there is a continuous search to optimize the process/solvent system to improve cost and extraction yield [157,171,173]. Ionic liquids have been extensively used for biomass dissolution and extraction, and the focus is to have a greener extraction process [113,114,115]. DESs have also been used and have shown extraction properties for multiple biomolecules such as phenolic compounds, flavonoids, and alkaloids [156,170,171,172,173,174,175,176]. Recently, Wu et al. (2021) [171] used DESs to extract polysaccharides from lotus leaves. A choline chloride- and ethylene glycol-based DES was used, and the extraction process was optimized and compared with the conventionally used hot water treatment [171]. The maximum extraction yield of 5.38% of lotus leaf polysaccharides was obtained, which was higher (3.22%) than that using hot water extraction [171]. The method demonstrated potential for extraction of acidic polysaccharides [171].

Polysaccharides can be extracted using hot water; however, such extraction is time consuming and requires a lot of energy [157]. High-temperature treatment is applied, and ethanol is used as a solvent to extract polysaccharides [156]. The biopolymer has been extracted using DES from various raw materials such as date palm biomass, corncob biomass, lotus leaves, rice straw, bulbs of *Lilium lancifolium*, edible fungus (*Poria cocos*), polyculture biofilms, etc. [157,171,172,173,174,177]. Strong DES–biomolecule interactions (hydrogen bonding and electrostatic) were responsible for extraction [157,173].

Polysaccharide from *Poria cocos* was extracted using DES composed of sorbitol, 1,3-butanediol, and choline chloride [173]. The DES extraction was assisted by far infra-red (FIR) radiation and thermal treatment [173]. The mixture of DES and *poria cocos* powder was irradiated with FIR and provided thermal energy from vortex airflow (hot air) [174]. The hot air was supplied for easy penetration of DES into the powdered *poria cocos* material [173]. The polysaccharide and DES were separated using dialysis and evaluated for recyclability of DES, and the recovery rate was found to be 80.69 ± 1.03% after five cycles of the recovery process [173]. The extracted polysaccharide was characterized using spectroscopic techniques (FT-IR, NMR). It was reported that the optimized extraction process using DES was found to be 4–70 times higher than the traditionally used extraction process [173].

NADES have also been explored to extract biomolecules and were recently used for a biofilm structure breaker [174]. A group of micro-organisms (fungi and/or bacteria) that were surrounded by an extracellular matrix formed of DNA and polymeric (polysaccharides) compounds form the biofilm [174,175]. This biofilm provided protection and is due to the polymeric extracellular substances, so treatment of biofilms involves the disruption of the polymeric compounds [174,175]. For complete removal of biofilm, chemicals are employed, which are a cause of environmental pollution [174,175]. Several NADES were used for the cleaning of biofilms [174,175]. The lactic acid–glucose–water and betaine–urea–water NADES were used as a green solvent system for a structural weakening of biofilm and were found to solubilize ≈ 70% of the main biofilm components [174]. Thus, the search is to find environmentally friendly solvent systems not only for extraction but also for the reusability of the solvent system, which can extract selectively with high efficiency valuable bioactive compounds.

#### 4.3.3. Proteins

Another application of DES solvent is for protein extraction [158,176,177]. The molecular part of life present in all cells and parts of the body, proteins have been extensively studied [156,175,176]. Due to their requirement in pharmaceutical and medicinal industries, protein extraction and purification have generated global interest. Sustainable protein production is required to reduce the pressure of producing, processing, or distributing food for the growing world population [177]. Protein consumption has also risen, and sustainable protein production and the search for new protein sources are ongoing [177]. Nutritional plant protein can be obtained once a high protein yield is selectively extracted from various plant-based sources [176,177]. Protein stability is vital, as proteins are denatured under alkaline and acidic environments or high-temperature exposure [176,177]. Protein purification and separation are thus challenging, and various techniques have been employed to overcome various limitations associated with protein extraction, separation, partition, or stability [176,177]. To avoid the denaturation of proteins, a new solvent system based on DES is being explored. The DES aqueous biphasic system (ABS) is also used for protein extraction [157,177].

Xu et al. [178] have used choline-based DES bovine serum albumin (BSA) extraction via ABS [178]. The extraction process was optimized by considering various parameters such as pH, the concentration of DES, a mass of protein, salt concentration, time, and temperature [178]. Spectroscopic analysis indicated that the protein conformation was preserved even after extraction in the DES phases [178]. The aggregate formation was confirmed from dynamic light scattering, conductivity, and TEM analysis, and the DES-protein agglomeration was reported to be the driving force for protein extraction. Various choline-based DESs were prepared using hydrogen acceptors such as D-glycose, glycerol, D-sorbitol, and ethylene glycol [178]. In optimized conditions, 98.15% of BSA was extracted into the DES rich phase [178].

Choline-based DES was also used in extracting vegetable protein from natural sources oilseed cakes. Water was used as an anti-solvent for forming protein-rich precipitates, and the methodology can extract 40–50% protein [179]. The protein extracted was confirmed from various spectroscopic techniques such as NMR, ATR-IR, CHN analysis, and TGA [178]. Li et al. [180] prepared betaine-based DESs and used them for bovine serum albumin, ovalbumin, and trypsin extraction [180]. Circular dichroism (CD), UV–visible, and FT-IR analysis were performed to understand the extraction mechanism. Further, TEM and DLS analyses were carried to study the extracted protein′s morphology [181].

**Figure 8:**
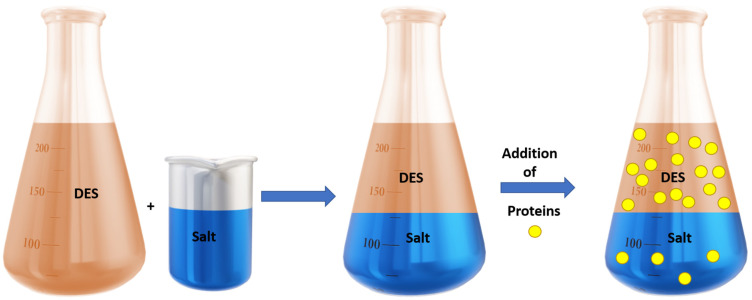
The protein extraction using an aqueous biphasic system (ABS) containing DES.

The DES-based ABS has been used extensively for protein extraction from natural sources [179,180,181,182,183,184]. The extraction with DES-ABS is depicted in Figure 8 [181].

DES-based aqueous biphasic system (ABS) with highly concentrated salt solution, as shown in Figure 8 [181]. The extraction of protein is performed with ABS system to prevent protein denaturation and preserve protein biological activity [182]. The mechanism for extraction is based on the fact that the competition between high salt concentration and protein occurs for water molecules [181]. The water content decreases for protein solubilization due to high salt concentration, causing protein concentration to be increased in DES [181]. Protein conformation remains maintained during extraction, and this was confirmed from spectroscopic analysis (UV–Visible and FT-IR) and CD measurements. Further, there is lack of interference between DES and BSA, and protein extraction is mainly governed by protein aggregation (i.e., surrounded by DES) [178,179,180,181,182,183,184]. The extraction of various biomolecules using DESs are shown in Table 7. Thus, DESs have shown potential as extracts of biomolecules ranging from flavonoids to proteins.

**Table 7:** The various biomolecules extracted using DESs.

DES	Flavonoids	Proteins	Polysaccharide Source	Ref.
ChCl:betaine hydrochloride:ethylene glycol	Genkwanin	-	-	[165]
ChCl:betaine hydrochloride:ethylene glycol	Apigenin	-	-	
ChCl:betaine hydrochloride:ethylene glycol	Quercetin-3-*O*-β-d-glucopyranoside	-	-	
ChCl:betaine hydrochloride:ethylene glycol	Kaempferol-3-*O*-β-d-glucopyranoside-7-*O*-β-d-glucopyranoside	-	-	
ChCl:betaine hydrochloride:ethylene glycol	Luteolin	-	-	
ChCl:betaine hydrochloride:ethylene glycol	Luteolin-7-*O*-β-d-glucopyranoside	-	-	
ChCl:betaine hydrochloride:ethylene glycol	Genkwanin-5-*O*-β-d-glucopyranoside	-	-	
ChCl:betaine hydrochloride:ethylene glycol	Apigenin-5-*O*-β-d-glucopyranoside	-	-	
ChCl:citric acid	Rutin	-	-	[164]
ChCl:glycerol		-	-	
ChCl:glucose:water		-	-	
ChCl:fructose:water		-	-	
ChCl:lactic acid	Wogononin	-		[166]
ChCl:lactic acid	Baicalein	-		
ChCl:lactic acid	Wogonoside	-		
ChCl:Sorbitol:1,3-butanediol	-	-	*Poria cocos* polysaccharides	[173]
Lactic acid:glucose:water	-	Bovine Serum Albumin	-	[174]
Betaine:urea:water		Bovine Serum Albumin	-	[174]
ChCl:ethylene glycol	-	-	*Lilium lancifolium* Thunb.	[184]
ChCl:ethylene glycol	-	-	Lotus leaves	[171]
ChCl:1,3-butanediol:D-sorbitol	-	-	*Poria cocos* (Schw.)wolf	[173]
ChCl:glycerol	-	Proteins from Rapeseed Cake and Primrose Cake	-	[179]
ChCl:ethylene glycol ChCl:glycerol ChCl:Sorbitol	-	Bovine Serum Albumin and Trypsin	-	[183]
Betaine–urea	-	Bovineserumalbumin, Ovalbumin, Trypsin	-	[180]
Sodium acetate:urea Potassium formate:urea	-	Brewer’s Spent Grain Proteins	-	[177]

## 5. Conclusions and Future Perspective

Volatile organic solvents (VOCs) are used extensively due to their widespread industrial and scientific applications. Several advantages and disadvantages are associated with VOCs. Considering the environmental issue, the search for replacement solvents has led to the synthesis, the characterization, and evaluating physicochemical properties and applications of neoteric solvents [88,183]. Research and development are ongoing of sustainable biocompatible neoteric candidates that can be synthesized with ease and have required/desired physicochemical properties besides satisfying the green chemistry principle.

Eutectic mixtures, DES, THEDES, and NADES are recent examples of biocompatible solvent systems [183], and they can be prepared using biocompatible starting materials described as HBAs and HBDs. One of the promising applications of these low melting liquids is in the pharmaceutical industry, as pharma industries use VOCs for drug synthesis, solubilization, and drug delivery/formulations [88,183]. Recently, the eutectic mixtures have been evaluated for various applications, and’ The uses of DEss in pharmacological preparations/drug solubilization and biomolecule extraction have been described in this review [88,183]. The choice of HBAs and HBDs provides an opportunity for making a safer solvent that can be a suitable solubilization media for APIs [88,183]. DES formulations (THEDES) have been tested on animal models, and recently, clinical translation for the treatment of rosacea has been undertaken [124]. For extraction, DESs have been employed in ABS for high yield extraction of various biomolecules from natural or waste materials. The nature of interactions between DES and molecules of interest (API, biomolecules) has been evaluated to understand the underlying mechanism of extraction. Nonetheless, research has been performed at a lab scale and, for industrial application, must be translated to a large scale. Evaluation of physicochemical properties such as density, viscosity, thermal stability, along with bio-profiles to evaluate the safety and health-related impacts of such a eutectic system, is required [183]. Detailed research is also needed to assess the molecular interactions fully, and such interactions can also be studied computationally along with experimental data [183]. Furthermore, the recyclability of such bio-compatible solvents must also be evaluated. The effect of long-term storage on DES or its formulation with API can be studied.
